# Ultra-Short-Term Wind Power Forecasting Based on CGAN-CNN-LSTM Model Supported by Lidar

**DOI:** 10.3390/s23094369

**Published:** 2023-04-28

**Authors:** Jinhua Zhang, Zhengyang Zhao, Jie Yan, Peng Cheng

**Affiliations:** 1 School of Energy and Power Engineering, North China University of Water Resources and Electric Power, Zhengzhou 450045, China; 2 College of New Energy, North China Electric Power University, Beijing 100096, China

**Keywords:** wind power forecasting, conditional generative adversarial network, convolutional neural network, long-short term memory, attention mechanism

## Abstract

Accurate prediction of wind power is of great significance to the stable operation of the power system and the vigorous development of the wind power industry. In order to further improve the accuracy of ultra-short-term wind power forecasting, an ultra-short-term wind power forecasting method based on the CGAN-CNN-LSTM algorithm is proposed. Firstly, the conditional generative adversarial network (CGAN) is used to fill in the missing segments of the data set. Then, the convolutional neural network (CNN) is used to extract the eigenvalues of the data, combined with the long short-term memory network (LSTM) to jointly construct a feature extraction module, and add an attention mechanism after the LSTM to assign weights to features, accelerate model convergence, and construct an ultra-short-term wind power forecasting model combined with the CGAN-CNN-LSTM. Finally, the position and function of each sensor in the Sole du Moulin Vieux wind farm in France is introduced. Then, using the sensor observation data of the wind farm as a test set, the CGAN-CNN-LSTM model was compared with the CNN-LSTM, LSTM, and SVM to verify the feasibility. At the same time, in order to prove the universality of this model and the ability of the CGAN, the model of the CNN-LSTM combined with the linear interpolation method is used for a controlled experiment with a data set of a wind farm in China. The final test results prove that the CGAN-CNN-LSTM model is not only more accurate in prediction results, but also applicable to a wide range of regions and has good value for the development of wind power.

## 1. Introduction

In recent years, as traditional non-renewable energy sources have been increasingly exhausted due to continuous utilization, emerging renewable clean energy sources have flourished under the strong support of the state, and wind power has developed particularly rapidly, with installed capacity increasing year by year. By the end of September 2022, the cumulative installed capacity of wind power in the country was 348 million kilowatts, an increase of 17% year-on-year, of which the newly installed wind power installed capacity reached 5.339 million kilowatts, an increase of 0.72% year-on-year, and the power generation increased steadily. During the first three quarters of 2022, the national wind power generation reached 158.1 billion kilowatt-hours, an increase of 26.5% year-on-year, accounting for 13.8% of the total power generation, and has become an important part of the national energy [1]. However, as the proportion of wind power continues to increase, the randomness and volatility of wind power generation can easily lead to insufficient or redundant output, the large-scale integration of wind power into the power grid will greatly increase the difficulty of making power generation plans, and the frequency and voltage of the power grid will fluctuate, which will have a negative impact on the internal operation of the power system [2], thereby restricting the developmental scale of wind power. Through the prediction results of the wind power forecasting system, the power grid dispatching department can reasonably arrange the power generation plan, reduce the spinning reserve capacity of the system, and improve the economy of the power grid operation. At the same time, by predicting the fluctuation of wind power in advance, the operation mode and countermeasures can be reasonably arranged. Therefore, accurate forecasting of wind power is of great significance to the stable operation of the power system [3].

The current wind power prediction methods are roughly divided into physical methods [4], statistical methods, and comprehensive methods [5,6]. The use of physical methods is based on the parameterization of detailed descriptions of atmospheric physics, generally referring to numerical weather prediction (NWP) by solving mathematical models such as temperature, pressure, and surface information [7,8]. Since the wind speed has strong randomness, intermittence, and volatility, how to accurately predict the wind speed change is also very important. Mohammed Bou-Rabee’s team used a merger of the artificial neural network (ANN) and the particle swarm optimization (PSO) model to accurately predict wind speed changes for the next month [9]. The statistical method is to predict the power of wind power through the statistical analysis of historical data and compare and adjust it with the recent measured power. Commonly used methods include the BP neural network method [10], the autoregressive moving average method [11], and the fuzzy logic method [12]. If physical and statistical methods are combined, it becomes a comprehensive method. The combined form is usually a combination of physical and statistical methods, short-term and medium-term prediction models, etc., which can effectively reduce errors and improve prediction accuracy, such as a hybrid integrated CFD and Kalman filter [13], a hybrid Fourier transform and wavelet transform [14], and a multi-algorithm hybrid [15]. These traditional power-generating prediction methods are to establish a model of the generating system first, and then use the power curve of the fan to calculate the actual output power of the generator. The advantage is that it does not require huge historical data for support, and it can be combined with the weather forecast model for prediction. However, the error of this method is large, and the stability of the prediction results is poor.

The deep learning algorithm is a neural network that establishes and simulates the human brain for analysis and learning [16]. In terms of wind farm power prediction, the deep learning algorithm collects a large number of data features, continuously updates and iterates, and self-trains, and finally, the output is optimal. The scheme greatly improves the accuracy and stability of prediction [17]. In [18], it is based on the long short-term memory network (LSTM), deep learning optimization technology, variational mode decomposition (VMD) [19], an LSTM-based wind power interval prediction model is realized. In [20], it combines the high-quality data feature extraction ability of the convolutional neural network (CNN) with the ability of the LSTM to describe the time series and obtains a more accurate prediction model. In [21], it proposes an LSTM neural network based on the attention mechanism, enabling the model to pay more attention to important information in the time series.

However, when encountering data sets with severe data loss or small samples, the models built by existing deep learning algorithms often have low prediction accuracy. As an emerging algorithm in recent years, GAN has been widely welcomed by people for its powerful learning ability. Through repeated confrontation training between the generator and the discriminator, the generated data are enough to make the real ones, and it has a wide range of applications in many fields. In [22], it proposed to use generative adversarial networks (GAN) to extract the characteristics of time and space in the process of wind/solar power generation, and suggested to improve the algorithm by combining Wasserstein distance. In [23], while introducing FSL, an improved GR-GAN algorithm is used to generate high-quality images while ensuring consistency between text and generated images.

Summarizing the various viewpoints above, this paper proposes a wind power prediction method based on the CGAN-CNN-LSTM. This method first uses the CGAN network to preprocess the historical wind power data, and then puts the sorted data into the CNN-LSTM model. Among them, the features are first extracted by the CNN convolutional neural network, and then the feature data are put into the LSTM time series for wind power prediction. The advantage of this model is that the CGAN network is used to generate and fill in the missing values of the data, and at the same time, the attention mechanism is added to the LSTM neural network to speed up the convergence of the model.

The main contributions of this study are summarized below:

- Proposed an ultra-short-term wind power forecasting model based on the CGAN-CNN-LSTM algorithm and verified its feasibility;
- Used GAN’s data supplement function to solve the problem of missing data in the original data set;
- The time scale of ultra-short-term wind power prediction is shortened to 5 min, which improves the prediction accuracy.

The rest of the paper is organized as follows. Section 2 mainly introduces the basic principles of each algorithm used in this model, including formulas and structural diagrams. In Section 3, the various algorithms mentioned above are combined into a complete prediction model, and the data supplement function of the model for incomplete data sets is emphasized, and then the operation of the CGAN-CNN-LSTM wind power prediction model is introduced in detailed steps. Section 4 is the calculation example verification. First, we introduced the layout of the Sole du Moulin Vieux wind farm in France, especially the sensors scattered around the wind turbines. Then, using the data set obtained by lidar, the four months of February, May, August, and November were selected as representatives of the four seasons of the year. We selected four machines in the wind farm for one month as test targets, comparing this model with the CNN-LSTM wind power forecasting model for multiple rounds. It was verified that the forecasting accuracy of this model is better through the line chart and evaluation function. Finally, Section 5 summarizes the full text, discusses the results, and explains the research significance of this model.

## 2. Introduction to Algorithm Theory

In order to facilitate the understanding of professional words and special words, this paper includes an abbreviation table, as shown in Table 1.

### 2.1. Generative Adversarial Network

**Figure 1:**
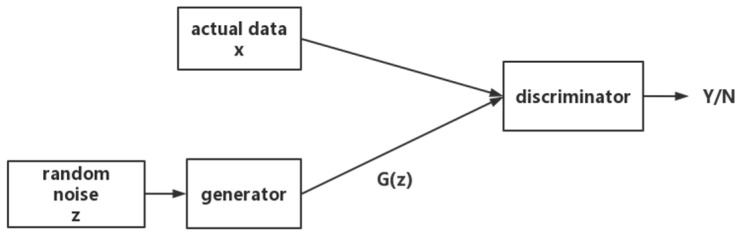
Generative adversarial network flowchart.

The generative adversarial network is a deep learning model [24], which produces a fairly good output in mutual game learning through two modules in the framework: the generative model and the discriminative model [25,26]. The random noise z is introduced into the generator, and the distribution of the mapping function is recorded as Pg(z). The discriminator receives the data from the generator Pz and the data of the real data Pdata at the same time, and outputs 0 or 1 to distinguish the generator. Whether the sample is true or not, the generator needs to make the output distribution converge to the real data distribution, and fool the discriminator, and the two adopt a confrontational method to improve each other’s performance. The process is shown in Figure 1:

The objective function of the discriminator D is:

(1) EDmaxX∼Pdata(X)[logD(X)]+Ez∼Pz(z)[log(1−D(G(z)))]

In the formula: $E_{X∼Pdata(X)}[\log D(X)]$ represents the value of the real data and represents the value of the data generated by the generator. The larger the sum of the two results, the better the effect of the discriminator.

The objective function of the generator G is:

(2) EGminz∼Pzlog(1−D(G(z)))

Since G(z) is a generated data distribution, the total objective function can be changed to:

(3) VG      Dmin max(D,G)=EX∼Pdata[logD(X)]+Ez∼Pzlog(1−D(x))

In the formula: VDmax(D,G) is to maximize the value function of the discriminator. The discriminator outputs 1 for real data and 0 for fake data. $\min_{G}$ is the value function to minimize the discriminator device; that is, it is hoped that D(X) is close to 0, and the result indicates that the generated data distribution is close to the real data distribution.

Since the image generated by the original GAN algorithm is random and unpredictable, the generated target is not clear, the controllability is not strong, and there are certain limitations. This paper chooses to use the conditional generative adversarial network (CGAN) [27,28]. The method of the CGAN is constructed by adding label(y) to the data, and additional conditional information is added to the input of the discriminator and the generator, which constrains the data generated by the generator [29]. Only data that is sufficiently real and qualified can be recognized by the discriminator. The objective function of the CGAN is:

(4) VG      Dmin max(D,G)=EX∼Pdata(X)[logD(X∣y)]+Ez∼Pz(z)[log(1−D(G(z∣y)))]

In the formula: D(X|y) and G(z|y) represent the condition *y* introduced by the CGAN, and the characteristic distribution of the sample X is calculated when the condition y is known.

### 2.2. Convolutional Neural Network

The CNN is a feed-forward neural network with a convolutional structure, which consists of an input layer, a convolutional layer, a pooling layer, and a fully connected layer [30,31]. It has a wide range of applications in image recognition, natural language processing, and remote sensing science. Compared with the traditional multi-layer neural network, the CNN adds a convolutional layer and a pooling layer [32] to the fully connected layer, which is more effective in extracting the feature learning part. The formula for the feature extraction of a one-dimensional convolution for the time series is:

(5) Y=σ(W×T+b)

In the formula: Y is the extracted feature; σ is the sigmoid activation function; W is the weight matrix; T is the time series; b is the bias vector.

### 2.3. Attention-LSTM Network

**Figure 2:**
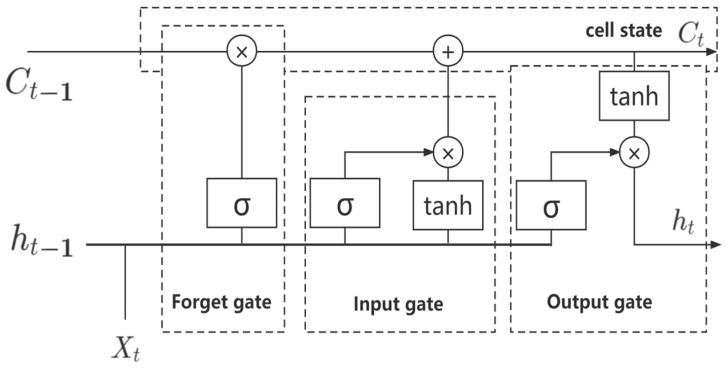
LSTM structure diagram.

LSTM is an efficient RNN architecture, which overcomes the problems of gradient disappearance and gradient explosion produced by RNN networks when dealing with long-term dependency problems [33]. The core concept of LSTM is cell state and “gate” structure, and each LSTM unit is composed of cell state, forget gate, input gate, and output gate. The LSTM structure is shown in Figure 2.

Set the input time series as $x=\{x_{1},x_{2},...,x_{t}\}$, and the two output sequences after LSTM mapping are $h=\{h_{1},h_{2},...,h_{t}\}$ and $y=\{y_{1},y_{2},...,y_{t}\}$. The forget gate in the LSTM unit decides which information should be discarded or retained, and its formula is:

(6) $$f_{t}=\sigma (W_{f}\times [h_{t-1},x_{t}]+b_{f})$$

In the formula: σ represents the sigmoid function; W and b are the parameters of the training network. The forget gate reads the previous output $h_{t-1}$ and the current output $x_{t}$, and then processes the sigmoid function to obtain the output $f_{t}$. The output value is between 0 and 1. If it is close to 0, it will be deleted, and if it is close to 1, it will be retained.

The input gate is to determine what kind of new information is stored in the cell state, which consists of two steps, and its formula is:

(7) $$i_{t}=\sigma (W_{i}\times [h_{t-1},x_{t}]+b_{i})$$

(8) $${\overset{\sim }{C}}_{t}=\tan h(W_{C}\times [h_{t-1},x_{t}]+b_{C})$$

In the formula: ${\overset{\sim }{C}}_{t}$ represents the new vector created by the tanh layer. The input gate obtains the data processed by the two functions of sigmoid and tanh, respectively, and combines the two into the cell state.

The cell state is to update $C_{t-1}$ to $C_{t}$; the formula is:

(9) $$C_{t}=f_{t}\times C_{t-1}+i_{t}\times {\overset{\sim }{C}}_{t}$$

The output gate determines what value needs to be output in the end; the formula is:

(10) $$o_{t}=\sigma (W_{o}\times [h_{t-1},x_{t}]+b_{o})$$

(11) $$h_{t}=o_{t}\times \tan h(C_{t})$$

It can be seen from the formula that the input data processed by the sigmoid function is multiplied by the cell state data processed by the tanh function, and the final data obtained is the output part.

**Figure 3:**
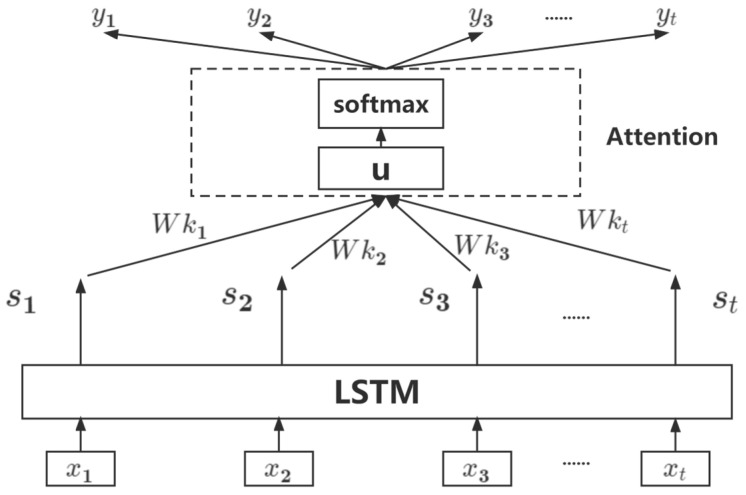
Attention-LSTM structure diagram.

The attention mechanism is derived from the study of human vision. Humans are used to selecting key parts of all information to remember, while forgetting other information [34]. For this prediction model, in order to make it focus more on the key information in the sequence, this paper adopts an attention-LSTM model, as shown in Figure 3.

After adding the attention mechanism, the output of each step of LSTM is calculated simultaneously with the current output, and finally the softmax function is used to generate a probability value. In Figure 3, after the input sequence $x_{1},x_{2},x_{3},...,x_{t}$ passes through the LSTM unit, the output sequence $s_{1},s_{2},s_{3},...,s_{t}$ is obtained. $Wk_{i}$ is the attention weight of each feature, and u is the feature representation. After attention processing, the output sequence $y_{1},y_{2},y_{3},...,y_{t}$ is obtained. The formula of the attention mechanism is as follows:

(12) $$B_{i}=\frac{\exp(Hk_{i})}{\sum_{i=1}^{t}\exp(Hk_{i})},\sum_{i=1}^{t}Hk_{i}=1$$

(13) $$Hk_{i}=u\tan h(Wk_{i}s_{i}+b_{i})$$

(14) $$u=\sum_{i=1}^{t}B_{i}s_{i}$$

In the formula: $B_{i}$ is the probability distribution value of each attention, $Hk_{i}$ is the attention mechanism matrix, and b is the bias. Finally, use the softmax function to obtain the predicted data $y_{t}$; the formula is as follows:

(15) $$y_{i}=soft\max(Wk_{i}u+b_{i})$$

## 3. Ultra-Short-Term Wind Power Forecasting Based on CGAN-CNN-LSTM

### 3.1. CGAN-CNN-LSTM Prediction Model

**Figure 4:**
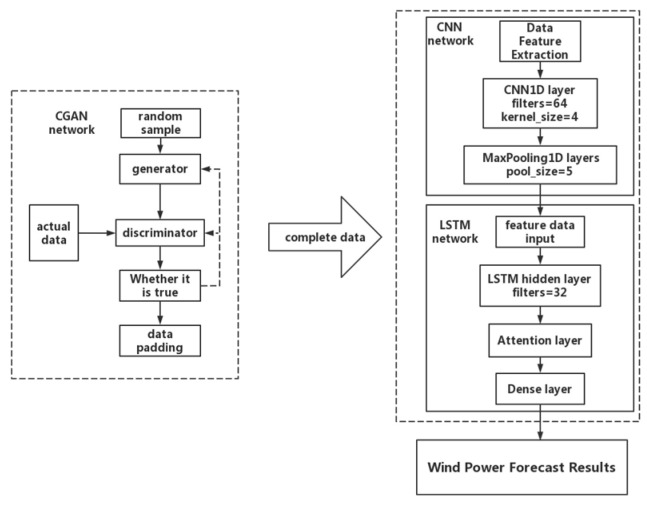
Ultra-short-term wind power forecasting model based on CGAN-CNN-LSTM.

Figure 4 is the structural diagram of the CGAN-CNN-LSTM wind power prediction model, which is divided into two stages: data processing and power prediction.

In the data processing stage, firstly, random samples enter the generator to generate data and are then input into the discriminator at the same time as the real samples. The discriminator is responsible for judging the authenticity of the two sets of data and uses the output to reversely update the generator and the discriminator. Under the continuous confrontation and updating of the generator and the discriminator, the data from the generator are finally very close to the data distribution of the real sample, and the discriminator cannot distinguish the real sample from the generated data. Finally, use the trained CGAN network to fill in the data [35].

In the power prediction stage, the CNN-LSTM model is selected, and the CNN local feature extraction module is a Conv1D layer, the number of convolution kernels is 64, and the size is 4; Followed by a batch normalization layer and a Maxpool1D layer. In LSTM, an attention module is added. The complete data are first input into the CNN network; then perform a local feature extraction and normalize the data. The formula is as follows:

(16) $$x_{nor}=\frac{x-x_{\min}}{x_{\max}-x_{\min}}$$

(17) $$y_{nor}=\frac{y-y_{\min}}{y_{\max}-y_{\min}}$$

In the formula: $x_{nor}$ represents the normalized data of meteorological characteristics; $x_{\max}$ and $x_{\min}$ represent the maximum and minimum values of meteorological characteristics; $y_{nor}$ represents the normalized data of the original wind power; y represents the original wind power data; $y_{\max}$ and $y_{\min}$ represent the maximum and minimum values of the wind power data.

Put the normalized data into convolutional and pooling layers for local feature extraction and stacking, and then the data with extracted feature information are predicted in the LSTM. Finally, in order to ensure that the data have physical meaning, it is necessary to denormalize the prediction results after the prediction is completed. The formula is as follows:

(18) $$y_{dnor}^{*}=(y_{\max}-y_{\min})y^{*}+y_{\min}$$

In the formula: $y_{dnor}^{*}$ represents the dimensioned wind power sequence after denormalization; $y^{*}$ represents the predicted value of the wind power.

### 3.2. Missing Value Supplementation

When processing the data, it was found that due to various factors such as the failure of the data collection equipment, weather factors, or collection errors, there were obvious data missing, so we chose to use the trained CGAN network to fill in the missing data. The formula is as follows:

(19) $$\overset{-}{X}=G(\overset{\sim }{X},M,(1-M)⊗z)$$

(20) X^=M⊗X~+(1−M)⊗X−

In the formula: $\overset{-}{X}$ represents the filling value; $\overset{\sim }{X}$ represents the data vector containing the missing values; M represents the mask vector, which only takes a value between 0 and 1, and is used to prompt the position of the missing data; X^ represents the final complete data vector. The data in $\overset{-}{X}$ are used for places with missing data, and the data in $\overset{\sim }{X}$ are used for places without missing data.

**Figure 5:**
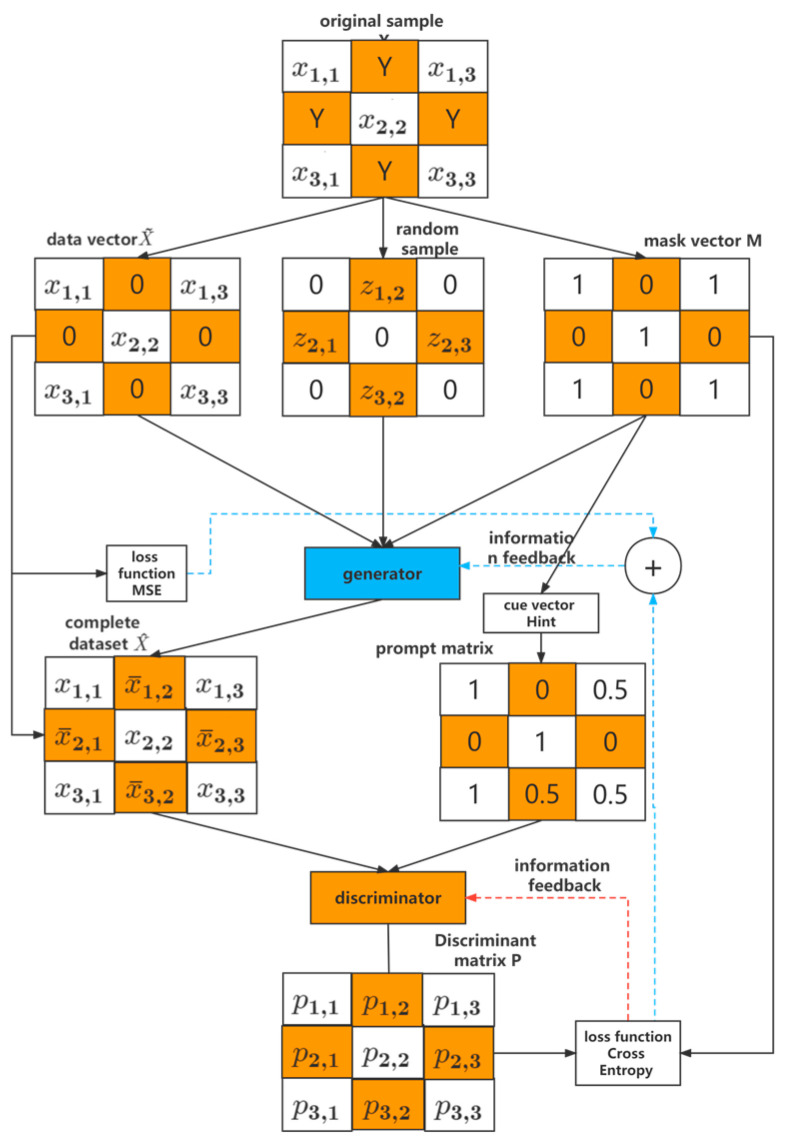
Missing value supplementary schematic.

The process of filling is to first remove the bad data in the original data set according to the missing rate and obtain the data vector $\overset{\sim }{X}$ containing missing values. Then, create a mask vector m that is equivalent to the size and dimension of $\overset{\sim }{X}$. Where there is data in $\overset{\sim }{X}$, M is represented by 1, and where there is no data, M is represented by 0. Input $\overset{\sim }{X}$, M, and random samples into the generator to obtain a new data set X^. While inheriting the original data of $\overset{\sim }{X}$, it also obtains new data $\overset{-}{X}$ through the generator and becomes a complete data set. Finally, input X^ and a hint vector, Hint, into the discriminator. After the same information feedback and confrontation optimization between the generator and the discriminator as before, the optimal solution is obtained, and a complete data set is output. The function of Hint is to carry out certain interference, to prevent the trained CGAN from being unable to continue training, and to speed up convergence at the same time. The principle of missing value supplementation is shown in Figure 5.

### 3.3. Wind Power Forecasting Process

Based on the three neural networks of CGAN, CNN, and LSTM, this paper constructs a combined model of the CGAN for data screening and supplementation and the CNN-LSTM for prediction, and adds an attention mechanism to the LSTM to accelerate the model convergence and improve wind power prediction accuracy. The prediction process is specifically divided into the following steps.

1. By importing the data set, it can be seen that a large section of data is missing. Through the CGAN, the data set is interpolated and filled to form a complete data set;
2. For the convenience of model calculation, the data are normalized; in order to make the data have physical meaning, the predicted results need to be denormalized;
3. Draw a heatmap to clearly see the correlation between the characteristic value and the wind power [36];
4. The data set is divided into a test set and a training set, and the output set is composed of time steps, respectively. After exchanging the rows and columns, the attention mechanism is applied to assign dynamic weights to the feature values;
5. By repeatedly training the model and comparing it with the test set, determine the group with the best evaluation function, and complete the establishment of the prediction model.

## 4. Example Verification

### 4.1. Lidar Wind Power Data Collection

At present, most wind farms use the method of building an anemometer tower to observe the wind conditions at the site continuously around the clock, and then record and store the measurement data in the data recorder installed on the tower body. However, the anemometer tower has disadvantages, such as many requirements for site selection, difficult maintenance, and high cost, which bring huge investment risks and loss of income to the construction, operation, and maintenance of wind farms. Lidar has the advantages of light and portable, easy installation, simple operation, and accurate wind measurement results. It is currently widely used in the field of wind power at home and abroad.

Wind Iris is the first wind turbine nacelle wind lidar developed by the French company Leosphere (Paris, France). It can measure the wind speed and wind direction in the range of 40~400 m directly in front of the hub of the wind turbine. Real-time data and statistics can be automatically transmitted via the data protocol or stored on the device itself. Wind Iris emits two laser beams at the same time to measure the wind speed at the hub height in front of the unit, and the wind speed measured by the two laser beams is processed to obtain the actual wind speed at the hub height at the measured position. However, Wind Iris cannot measure data such as wind direction, air pressure, temperature, and humidity.

The WindCube V2 land-based lidar is also developed by Leosphere (Paris, France). The wind speed, wind direction, and other indicators are measured by measuring the frequency change in the moving speed of the aerosol in the air by the pulsed laser. Measuring equipment such as air pressure, temperature, and humidity are embedded in the lidar wind measurement system. The WindCube V2 lidar emits four laser beams at the same time, measures the wind speed at four points within each layer height, performs weighted processing, and obtains the average wind speed and wind direction of the layer height.

Both lidars use laser pulse Doppler. The principle of frequency shift, by measuring the Doppler frequency shift generated by the aerosol backscatter echo signal; accurate real-time wind field data; and aerosol backscatter data are obtained to invert wind speed and wind direction information [37,38]. The specific working principle is as follows:

Let the laser-emitting module and the receiving module be used as the inertial system S, and the measured object be used as the inertial system A; the motion speed of the inertial system A relative to the inertial system S is V. When the laser source emits a beam of laser light with frequency $f_{0}$ to the measured object, in the inertial system A, the laser frequency at the measured object is:

(21) $$f_{s}=f_{0}+\frac{V}{\lambda }\cos\beta$$

The emitted laser light is backscattered by aerosol particles, and part of the light is reflected back to the detector. In the inertial system S, the laser frequency at the receiving module is:

(22) $$f_{r}=f_{s}+\frac{V}{\lambda }\cos\alpha$$

Therefore, there is a frequency difference between the local oscillator light and the echo signal, and the frequency difference is the Doppler frequency shift, namely:

(23) $$\Delta f_{d}=f_{r}-f_{0}=\frac{2}{\lambda }\frac{V(\cos\alpha +\cos\beta )}{2}$$

The above Doppler frequency shift formula is applicable to the off-axis coherent wind lidar system. For the transceiver coaxial system, the laser transmitting module and the receiving module are the same telescope, α=β=θ, Formula (23) can be simplified as:

(24) $$\Delta f_{d}=\frac{2V\cos\theta }{\lambda }$$

Among them, λ is the laser wavelength, V is the moving speed of the measured object, and θ is the angle between the measured object and the emitting laser.

### 4.2. Data Sources

The data set used in this paper is the measured data of Sole du Moulin Vieux (SMV) wind farm in France provided by ENGIE Green [39,40]. The data include NWP data and real-time wind power data of seven wind turbines. The wind farm is equipped with an advanced laser radar system and adopts a coordinated control strategy based on the coefficient of power (CP) [41]. Lidar is an important sensor used in surveying and mapping, mainly including ranging, positioning, and three-dimensional rendering of surface objects. In this wind farm, it is mainly used to collect data information such as wind speed, wind direction, and temperature. All turbines were equipped with a supervisory control and data acquisition (SCADA) system allowing 1 Hz data for the most critical variables to be recorded. SMV6 is equipped with Orion 5-beam laser radar for free-flow wind. A Vaisala Triton sodar was installed in the proximity of turbines SMV5 and SMV6, and a Leosphere (Paris, France) Windcube V1 ground lidar was installed between the SMV2 and SMV3 [42]. The radar measured the wind speed frequency at a height of 40 m to 200 m at a frequency of 1 Hz. Although data from the sodar and profiling lidar were not used extensively during the analysis, they were used to cross-check and validate wind measurements from the turbines and to identify the best references for assessing the ambient wind conditions. The scanning lidar was installed on the east side, 1.2 km away from the wind farm, and it can measure the hub height of SMV6 [43]. For the lidar-based analyses presented here, we used the estimated horizontal wind speeds and wind directions at hub height provided by the lidar.

### 4.3. Experimental Platform and Evaluation Criteria

Based on the Tensorflow 2.2 deep learning library in Python 3.7 software, a prediction model was built, and the Adma optimization algorithm was introduced to update the weight of the neural network in the form of data iteration; the number of iterations is 100. Finally, the evaluation functions root means square error (RMSE) and R-square ($R^{2}$) are used to judge the precision of the model; the formula is as follows.

(25) $$RMSE=\sqrt{\frac{\sum_{n}^{i=1}(Y_{i}-{\hat{Y}}_{i}{)}^{2}}{n}}$$

(26) $$R^{2}=1-\frac{\sum_{n}^{i=1}(Y_{i}-{\hat{Y}}_{i}{)}^{2}}{{\sum_{n}^{i=1}\left(Y_{i}-\overset{-}{Y}\right)}^{2}}$$

Root means square error (RMSE) is the square root of the ratio of the square of the deviation between the predicted value and the true value and the number of observations n, which is a typical indicator of the regression model. The smaller the RMSE, the better the prediction model. $R^{2}$ is the ratio of 1 minus the sum of the squares of the distances between all observations and predicted values to the sum of squares of the distances between all observations and the mean. The closer the $R^{2}$ value is to 1, the better the prediction model is.

### 4.4. Experimental Results and Model Comparison

**Figure 6:**
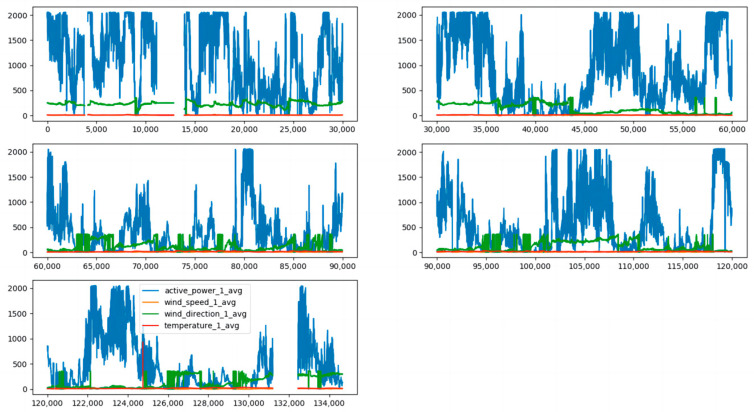
Missing data.

**Figure 7:**
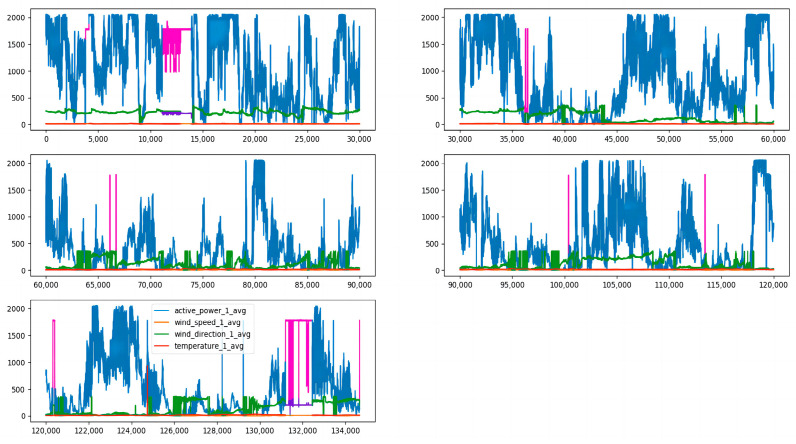
Missing data imputation.

Since the CGAN-CNN-LSTM model is relatively advanced, it is meaningless to compare it with the BP neural network, Elman neural network, and other prediction models, so the CNN-LSTM, LSTM, and SVM prediction models were selected for comparative experiments. First, we imported all the data sets. From Figure 6, it can be seen that the data in the first part and the data in the fifth part are obviously missing. Figure 7 shows the effect after supplementing the data with the CGAN correlation, data supplements have been highlighted in different colors.

**Figure 8:**
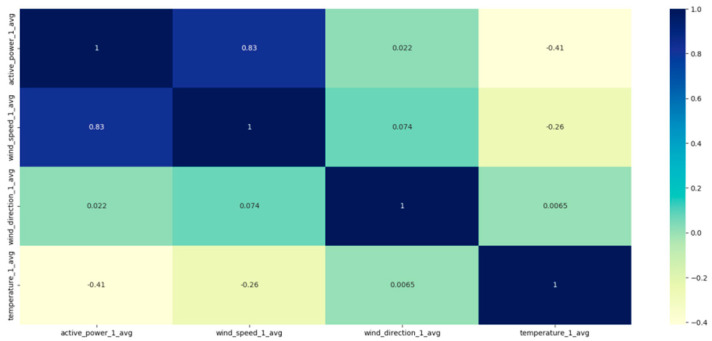
Heatmap.

**Figure 9:**
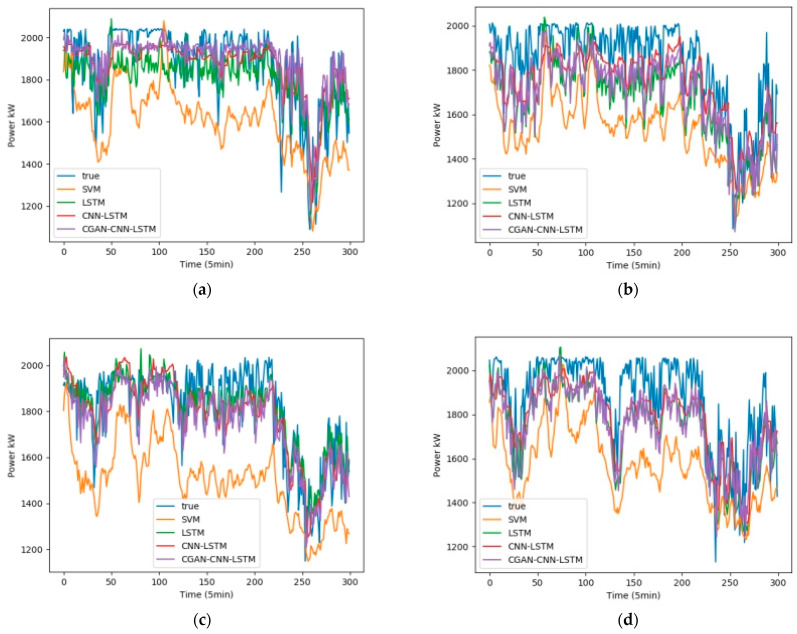
Wind power forecast results of 4 wind turbines in February (**a**) Power Forecast of SMV1; (**b**) Power Forecast of SMV3; (**c**) Power Forecast of SMV5; (**d**) Power Forecast of SMV7.

**Figure 10:**
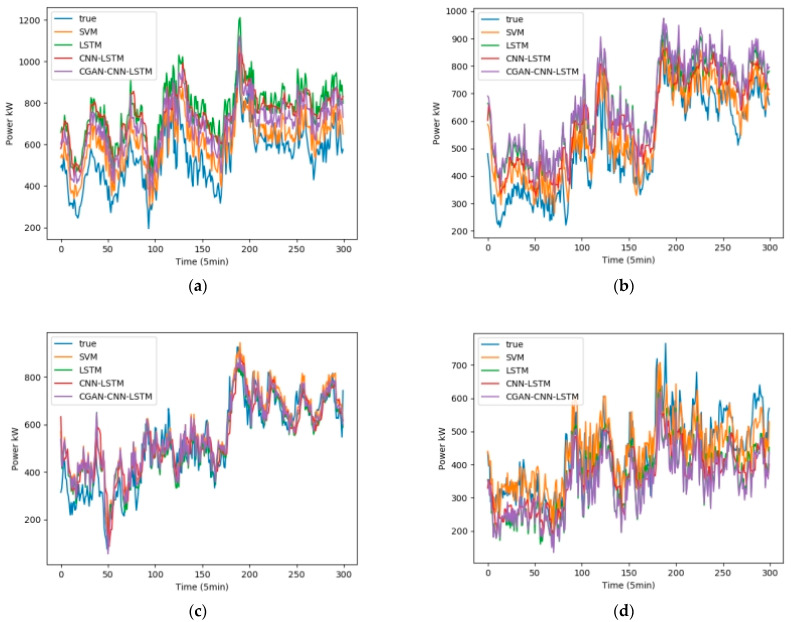
Wind power forecast results of 4 wind turbines in May (**a**) Power Forecast of SMV1; (**b**) Power Forecast of SMV3; (**c**) Power Forecast of SMV5; (**d**) Power Forecast of SMV7.

**Figure 11:**
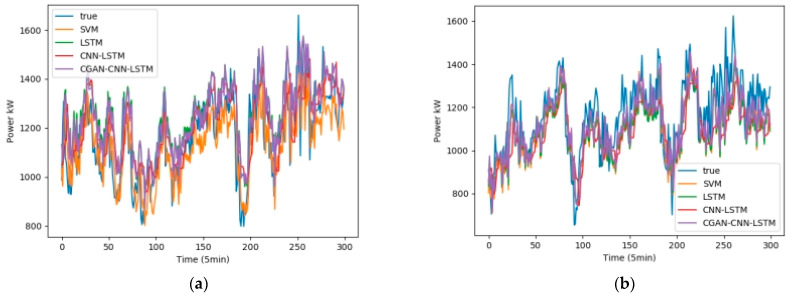

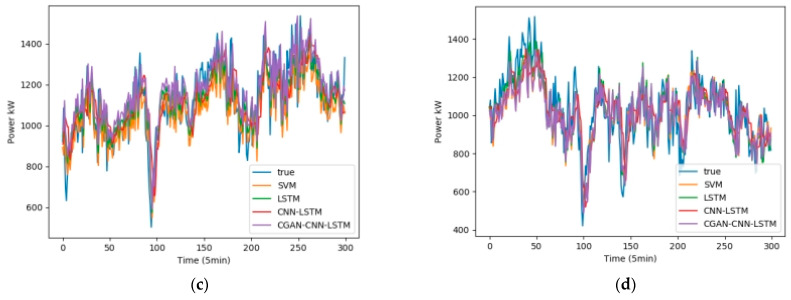
Wind power forecast results of 4 wind turbines in August (**a**) Power Forecast of SMV1; (**b**) Power Forecast of SMV3; (**c**) Power Forecast of SMV5; (**d**) Power Forecast of SMV7.

**Figure 12:**
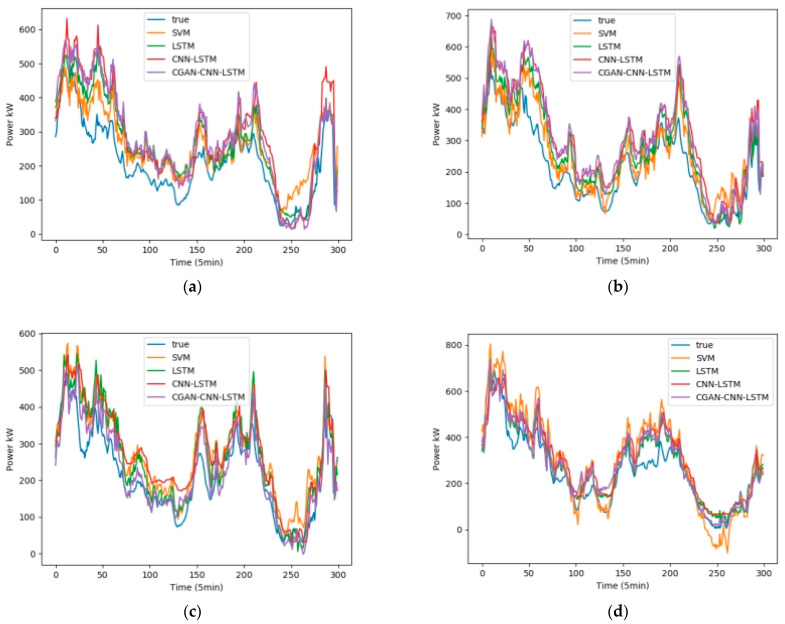
Wind power forecast results of 4 wind turbines in November (**a**) Power Forecast of SMV1; (**b**) Power Forecast of SMV3; (**c**) Power Forecast of SMV5; (**d**) Power Forecast of SMV7.

We selected February, May, August, and November from the one-year data set, 5 days per month as the training set, and 1 day as the test set. Fans 1, 3, 5, and 7 were selected as test units. The wind speed, wind direction, and temperature in the NWP data are used as the input data, and the output is the wind power. Figure 8 shows the influence of the wind speed, wind direction, and temperature on the wind power, among which wind speed has the greatest influence, and wind direction has the least influence. Figure 9 shows the test results in February, Figure 10 shows the test results in May, Figure 11 shows the test results in August, and Figure 12 shows the test results in November. In the simulation diagram, the 300 sampling points in the image are divided according to the time scale, and a sampling point every 5 min predicts the wind power of a day.

Table 2 shows the data of the two loss functions of $R^{2}$ and RMSE of the four forecasting models in February, and Table 3, Table 4 and Table 5 represent the same content in May, August, and September, respectively.

It can be seen from Table 2 that the average values of $R^{2}$ of the four prediction models of CGAN-CNN-LSTM, CNN-LSTM, LSTM, and SVM are 0.934, 0.922, 0.911, and 0.847; the average values of RMSE are 0.0804, 0.0871, 0.0939, and 0.1236, respectively.

It can be seen from Table 3 that the average values of $R^{2}$ of the four prediction models of CGAN-CNN-LSTM, CNN-LSTM, LSTM, and SVM are 0.911, 0.873, 0.888, and 0.891; the average values of RMSE are 0.0733, 0.0872, 0.0826, and 0.0783, respectively.

It can be seen from Table 4 that the average values of $R^{2}$ of the four prediction models of CGAN-CNN-LSTM, CNN-LSTM, LSTM, and SVM are 0.925, 0.912, 0.899, and 0.898; the average values of RMSE are 0.0710, 0.0739, 0.0785, and 0.0803, respectively.

It can be seen from Table 5 that the average values of $R^{2}$ of the four prediction models of CGAN-CNN-LSTM, CNN-LSTM, LSTM, and SVM are 0.911, 0.887, 0.881, and 0.892; the average values of RMSE are 0.0847, 0.1056, 0.1060, and 0.1001, respectively.

For the whole year, it can be seen from the figure that the forecasts for February and August are better than those for May and November. This is because February and August are windy months, with strong and relatively stable wind speeds, and less fluctuations in wind power power, which are easier to predict. It can also be seen from the figure that the fitting curves of SMV5 and SMV7 are obviously better than those of SMV1 and SMV3. This is because there are a small number of wind speed and temperature in the data sets of SMV1 and SMV3 units. Or the record of the wind direction is missing, which does not match the power value at the same time, resulting in some impact on the power prediction of the model. The final results show that for different wind turbines tested in different months in the same wind farm, The average values of $R^{2}$ of the four prediction models of CGAN-CNN-LSTM, CNN-LSTM, LSTM, and SVM are 0.921, 0.899, 0.895, and 0.882; the average values of RMSE are 0.0774, 0.0885, 0.0903, and 0.0956, respectively. Compared with the best CNN-LSTM in the control experiment, CGAN-CNN-LSTM increased $R^{2}$ by 2.45% and RMSE decreased by 12.5%. It proves that this model is more accurate in predicting wind power.

**Figure 13:**
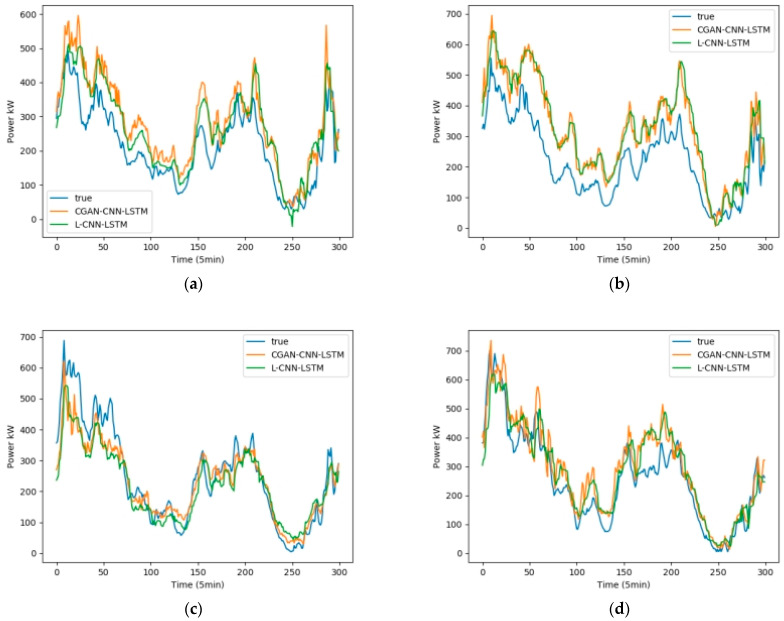
Wind power forecast results of 4 wind turbines in China’s Wind Farm in March (**a**) Power Forecast of machine 1; (**b**) Power Forecast of machine 2; (**c**) Power Forecast of machine 3; (**d**) Power Forecast of machine 4.

In order to show that the model is applicable to wind farms all over the world, and to verify the difference between CGAN and general interpolation methods, this experiment adds a set of control experiments in Chinese wind farms. The content of the experiment is to set the data of the wind farm from March 1st to 5th as the training set. The data on March 6 was set as the test set, and the CGAN-CNN-LSTM model and the CNN-LSTM with linear interpolation model (L-CNN-LSTM) were used to make predictions, and the test results of the four machines were compared, Figure 13 and Table 6 are the test results in March. Table 6 is the $R^{2}$ and RMSE evaluation functions of these two groups of models.

In experiments in wind farms in China, the final results show that the average $R^{2}$ value of the CGAN-CNN-LSTM prediction model is 0.927, and the $R^{2}$ average value of the L-CNN-LSTM prediction model is 0.887; The average RMSE value of the CGAN-CNN-LSTM prediction is 0.0812, and the average RMSE value of the L-CNN-LSTM prediction model is 0.0926. It can be seen that compared with the L-CNN-LSTM prediction model, the $R^{2}$ value of this model increases by 4.5% on average, and the RMSE value decreases by 12.3% on average, which is closer to the actual wind power curve.

## 5. Conclusions

In response to the increasing accuracy requirements of wind power forecasting, this paper proposes a combined forecasting model of CGAN-CNN-LSTM, select 4 typical monthly data of 4 units in the French wind farm as the data set for testing, and choose CNN-LSTM, LSTM, SVM as the comparison algorithm. The test result is compared with the best CNN-LSTM in the control experiment, CGAN-CNN-LSTM increased $R^{2}$ by 2.45% and RMSE decreased by 12.5%. It proves that this model is more accurate in predicting wind power. Then, in order to prove the universality of this model and the ability of the CGAN algorithm, a wind farm in China was selected as a data set and compared with L-CNN-LSTM. The results show that the $R^{2}$ value of this model increases by 4.5% on average, and the RMSE value decreases by 12.3% on average, which is closer to the actual wind power curve. It also shows that the model is applicable to different wind farms around the world. The main features of this model are as follows:

1. Use CGAN to fill in the missing data of the NWP dataset to obtain a complete dataset.
2. Use CNN to extract features from the data set, and then use LSTM algorithm to predict wind power.
3. The Attention mechanism is added to the LSTM algorithm to make the model pay more attention to the key information in the sequence, speed up the convergence speed, and improve the model accuracy.

It solves the problem of partial missing of the original data set, and provides new ideas and methods for improving the accuracy of ultra-short-term wind power prediction.

However, this model also has shortcomings. When filling in the missing data, it will be difficult to simulate real data if faced with data with large differences between before and after. In the GAN series, more excellent variants will be developed in the future, and models based on them will make wind power prediction more accurate.

## Figures and Tables

**Table 1:** Acronym list.

Acronym	Implication
GAN	Generative adversarial network
CGAN	Conditional generative adversarial network
CNN	Convolutional neural network
LSTM	Long short-term memory
CFD	Computational fluid dynamics
VMD	Variational mode decomposition
NWP	Numerical weather prediction
SMV	Sole du Moulin Vieux
SCADA	Supervisory control and data acquisition
SVM	Support vector machine

**Table 2:** Prediction errors of CGAN-CNN-LSTM and CNN-LSTM in February.

Machine	Model	$R^{2}$	RMSE
SMV1	CGAN-CNN-LSTM	0.914	0.0983
	CNN-LSTM	0.893	0.1041
	LSTM	0.884	0.1074
	SVM	0.837	0.1281
SMV3	CGAN-CNN-LSTM	0.954	0.0702
	CNN-LSTM	0.942	0.0831
	LSTM	0.929	0.0875
	SVM	0.864	0.1296
SMV5	CGAN-CNN-LSTM	0.925	0.0815
	CNN-LSTM	0.918	0.0842
	LSTM	0.906	0.0987
	SVM	0.830	0.1221
SMV7	CGAN-CNN-LSTM	0.944	0.0715
	CNN-LSTM	0.933	0.0771
	LSTM	0.923	0.0821
	SVM	0.856	0.1145

**Table 3:** Prediction errors of CGAN-CNN-LSTM and CNN-LSTM in May.

Machine	Model	$R^{2}$	RMSE
SMV1	CGAN-CNN-LSTM	0.926	0.0713
	CNN-LSTM	0.863	0.0966
	LSTM	0.887	0.0881
	SVM	0.903	0.0763
SMV3	CGAN-CNN-LSTM	0.911	0.0752
	CNN-LSTM	0.876	0.0866
	LSTM	0.901	0.0774
	SVM	0.892	0.0782
SMV5	CGAN-CNN-LSTM	0.905	0.0723
	CNN-LSTM	0.876	0.0834
	LSTM	0.894	0.0751
	SVM	0.883	0.0785
SMV7	CGAN-CNN-LSTM	0.901	0.0742
	CNN-LSTM	0.875	0.0821
	LSTM	0.869	0.0898
	SVM	0.884	0.0803

**Table 4:** Prediction errors of CGAN-CNN-LSTM and CNN-LSTM in August.

Machine	Model	$R^{2}$	RMSE
SMV1	CGAN-CNN-LSTM	0.906	0.0809
	CNN-LSTM	0.891	0.0826
	LSTM	0.872	0.0877
	SVM	0.883	0.0854
SMV3	CGAN-CNN-LSTM	0.932	0.0673
	CNN-LSTM	0.921	0.0691
	LSTM	0.914	0.0708
	SVM	0.905	0.0765
SMV5	CGAN-CNN-LSTM	0.928	0.0667
	CNN-LSTM	0.913	0.0736
	LSTM	0.893	0.0816
	SVM	0.903	0.0776
SMV7	CGAN-CNN-LSTM	0.933	0.0691
	CNN-LSTM	0.921	0.0705
	LSTM	0.915	0.0738
	SVM	0.901	0.0816

**Table 5:** Prediction errors of CGAN-CNN-LSTM and CNN-LSTM in November.

Machine	Model	$R^{2}$	RMSE
SMV1	CGAN-CNN-LSTM	0.895	0.0976
	CNN-LSTM	0.866	0.1222
	LSTM	0.882	0.1140
	SVM	0.887	0.1003
SMV3	CGAN-CNN-LSTM	0.921	0.0778
	CNN-LSTM	0.885	0.1045
	LSTM	0.897	0.0988
	SVM	0.895	0.0999
SMV5	CGAN-CNN-LSTM	0.915	0.0713
	CNN-LSTM	0.896	0.0958
	LSTM	0.909	0.0895
	SVM	0.901	0.0921
SMV7	CGAN-CNN-LSTM	0.912	0.0921
	CNN-LSTM	0.901	0.0997
	LSTM	0.834	0.1217
	SVM	0.884	0.1078

**Table 6:** Prediction errors of CGAN-CNN-LSTM and L-CNN-LSTM in China’s Wind Farm in March.

Machine	Model	$R^{2}$	RMSE
Machine 1	CGAN-CNN-LSTM	0.945	0.0754
	L-CNN-LSTM	0.906	0.0862
Machine 2	CGAN-CNN-LSTM	0.923	0.0845
	L-CNN-LSTM	0.885	0.0948
Machine 3	CGAN-CNN-LSTM	0.915	0.0863
	L-CNN-LSTM	0.876	0.0953
Machine 4	CGAN-CNN-LSTM	0.924	0.0784
	L-CNN-LSTM	0.881	0.0942

## Data Availability

The dataset used in this research has been obtained by ENGIE Green in the scope of French national project SMARTEOLE (grant no. ANR-14-CE05-0034).
